# Mitochondrial Fragmentation and Dysfunction in Type IIx/IIb Diaphragm Muscle Fibers in 24-Month Old Fischer 344 Rats

**DOI:** 10.3389/fphys.2021.727585

**Published:** 2021-09-28

**Authors:** Alyssa D. Brown, Leah A. Davis, Matthew J. Fogarty, Gary C. Sieck

**Affiliations:** Department of Physiology and Biomedical Engineering, Mayo Clinic, Rochester, MN, United States

**Keywords:** sarcopenia, fiber type, SDH_*max*_, maximum respiratory capacity, mitochondrial fragmentation

## Abstract

Sarcopenia is characterized by muscle fiber atrophy and weakness, which may be associated with mitochondrial fragmentation and dysfunction. Mitochondrial remodeling and biogenesis in muscle fibers occurs in response to exercise and increased muscle activity. However, the adaptability mitochondria may decrease with age. The diaphragm muscle (DIAm) sustains breathing, *via* recruitment of fatigue-resistant type I and IIa fibers. More fatigable, type IIx/IIb DIAm fibers are infrequently recruited during airway protective and expulsive behaviors. DIAm sarcopenia is restricted to the atrophy of type IIx/IIb fibers, which impairs higher force airway protective and expulsive behaviors. The aerobic capacity to generate ATP within muscle fibers depends on the volume and intrinsic respiratory capacity of mitochondria. In the present study, mitochondria in type-identified DIAm fibers were labeled using MitoTracker Green and imaged in 3-D using confocal microscopy. Mitochondrial volume density was higher in type I and IIa DIAm fibers compared with type IIx/IIb fibers. Mitochondrial volume density did not change with age in type I and IIa fibers but was reduced in type IIx/IIb fibers in 24-month rats. Furthermore, mitochondria were more fragmented in type IIx/IIb compared with type I and IIa fibers, and worsened in 24-month rats. The maximum respiratory capacity of mitochondria in DIAm fibers was determined using a quantitative histochemical technique to measure the maximum velocity of the succinate dehydrogenase reaction (SDH_*max*_). SDH_*max*_ per fiber volume was higher in type I and IIa DIAm fibers and did not change with age. In contrast, SDH_*max*_ per fiber volume decreased with age in type IIx/IIb DIAm fibers. There were two distinct clusters for SDH_*max*_ per fiber volume and mitochondrial volume density, one comprising type I and IIa fibers and the second comprising type IIx/IIb fibers. The separation of these clusters increased with aging. There was also a clear relation between SDH_*max*_ per mitochondrial volume and the extent of mitochondrial fragmentation. The results show that DIAm sarcopenia is restricted to type IIx/IIb DIAm fibers and related to reduced mitochondrial volume, mitochondrial fragmentation and reduced SDH_*max*_ per fiber volume.

## Introduction

The diaphragm muscle (DIAm) exhibits reduced specific force (force per cross-sectional area) and atrophy (decreased cross-sectional area) with aging, i.e., sarcopenia. In older (24 months) rats, DIAm fiber atrophy is restricted to type IIx/IIb fibers (Elliott et al., 2016; Khurram et al., 2018b; Fogarty et al., 2019b, 2020), which comprise more fatigable motor units (Sieck and Fournier, 1989; Sieck et al., 1989a; Sieck, 1991, 1995). Aging has no effect on type I and IIa fibers in the rat DIAm (Elliott et al., 2016; Khurram et al., 2018b; Fogarty et al., 2019b, 2020), which comprise fatigue resistant motor units that are recruited during breathing (Sieck and Fournier, 1989; Sieck et al., 1989a; Sieck, 1991, 1995).

It has been suggested that sarcopenia is triggered by mitochondrial fragmentation and dysfunction (Calvani et al., 2013; Marzetti et al., 2013). The aerobic capacity to generate ATP within muscle fibers depends on mitochondrial volume and the intrinsic respiratory capacity of mitochondria. Mitochondria are highly malleable and adapt to energy demand and oxidative stress (Aravamudan et al., 2017; Delmotte et al., 2017; Eisner et al., 2017; Delmotte and Sieck, 2019). This is evident by mitochondrial biogenesis in muscle fibers in response to exercise and increased muscle activity and the demands of homeostasis (Forman et al., 1987; Bereiter-Hahn and Voth, 1994; Prakash et al., 2017). Importantly, it has been reported that mitochondrial biogenesis decreases with age (Reznick et al., 2007; Ungvari et al., 2008). In the DIAm, type I and IIa fibers are very active being recruited to accomplish breathing with a duty cycle of ∼40% (Sieck and Fournier, 1989; Sieck, 1991, 1995; Khurram et al., 2018b). In contrast, type IIx/IIb DIAm fibers are far less active being infrequently recruited only during airway protective and expulsive behaviors with a duty cycle of <10% (Fogarty and Sieck, 2019a,b). Importantly, the ventilatory requirements for the recruitment of type I and IIa fibers continue in old age, whereas the selective atrophy and weakening of type IIx/IIb fibers impairs higher force airway protective and expulsive behaviors in rats (Fogarty et al., 2018b; Khurram et al., 2018b; Fogarty and Sieck, 2019b) and likely in humans (Enright et al., 1994; Tolep et al., 1995; Polkey et al., 1997; Fogarty and Sieck, 2019b). This reduced efficacy of airway defense maneuvers contributes to the increased incidence of respiratory morbidity and mortality in the aging cohort (Polkey et al., 1997; Mylotte et al., 2003; Fogarty et al., 2018a).

In a previous study, we used 2-D electron microscopic (EM) imaging of single type identified rat DIAm fibers to estimate mitochondrial volume densities. We found that there are marked differences in mitochondrial volume densities across rat DIAm fiber types reflecting differences in their activity (Sieck et al., 1998). The mitochondrial volume densities of type I and IIa DIAm fibers were found to be ∼10-fold greater than those of type IIx/IIb fibers (Sieck et al., 1998). However, this 2-D EM approach is greatly limited by the requirement for single fiber dissections, which yield very small sample sizes per fiber type. As an alternative, we recently, we developed and validated a 3-D confocal imaging technique based on MitoTracker Green labeling to quantify mitochondrial volume and morphology in airway smooth muscle cells (Aravamudan et al., 2014, 2017; Delmotte et al., 2017, 2020; Delmotte and Sieck, 2019), primary human skeletal muscle cells (hSkMCs) (Ryan et al., 2015), and phrenic motor neurons (Fogarty et al., 2021b). The present study used this confocal imaging technique to quantify mitochondrial volume and morphology in a large sample of type identified DIAm fibers.

To determine the maximum respiratory capacity of mitochondria in muscle fibers, we previously developed a quantitative histochemical technique to measure the maximum velocity of the succinate dehydrogenase reaction (SDH_*max*_) (Sieck et al., 1986, 1995, 1996; Blanco et al., 1988, 1995; Watchko and Sieck, 1993). Succinate dehydrogenase is a key enzyme in the tricarboxylic acid (TCA) cycle as well as complex II of the mitochondrial electron transport chain. Normalizing SDH_*max*_ to muscle fiber volume provides a measure of the total respiratory capacity of a muscle fiber, which we found is higher in type I and IIa DIAm fibers as compared with type IIx/IIb fibers (Sieck et al., 1986, 1995, 1996; Blanco et al., 1988, 1995; Watchko and Sieck, 1993). In the present study, we hypothesize that fiber type differences in SDH_*max*_ per muscle fiber volume are due to differences in mitochondrial volume density. Normalizing SDH_*max*_ to mitochondrial volume provides a measure of the intrinsic respiratory capacity of mitochondria, which we hypothesize also varies across fiber types due to differences in morphology (e.g., extent of mitochondrial fragmentation). The purpose of the present study was to examine age-related changes in mitochondrial morphology, mitochondrial volume density, and SDH_*max*_ in different DIAm fiber types. Overall, we hypothesize that sarcopenia related changes in mitochondrial structure and function are restricted to type IIx/IIb DIAm fibers.

## Materials and Methods

### Animals and Tissue Preparation

All protocols were approved by the Mayo Clinic Institute Animal Care and Use Committee (IACUC #A57714) and complied with National Institutes of Health (NIH) and American Physiological Society guidelines. Strips of diaphragm tissue were harvested from 24 pathogen-free 6- (young) and 24-month (old) Fischer 344 rats (6 month: 225–330 g; 24 month: 250–430 g; 12 females, 12 males; 6 months obtained from Charles River and 24 months from National Institute of Aging). Two rats per cage were housed under a 12:12 h light–dark cycle with *ad libitum* access to food and water. The animals were allowed at least 1 week to acclimatize to these conditions before experiments were performed. At the terminal experiment, animals were deeply anesthetized with intraperitoneal injection of ketamine (80 mg/kg) and xylazine (10 mg/kg) and euthanized *via* exsanguination. Following euthanasia, the DIAm was excised and two adjacent DIAm strips (∼2 mm width) were dissected from the mid-costal region and placed in Rees–Simpson solution (containing, in mM: 135 Na^+^, 5 K^+^, 2 Ca^2+^, 1 Mg^2+^, 120 Cl^–^, and 25 HCO_3_^–^) aerated with carbogen gas (95% O_2_–5% CO_2_) at room temperature. The first strip was used to measure DIAm specific force and fatigue, whereas the second DIAm strip was stretched to 1.5× resting length, which we previously found approximates optimal sarcomere length (2.5 μm) (Prakash et al., 1993a; Zhan et al., 1997). The muscle strip was then pinned to a piece of cork and rapidly frozen by submerging it in melting isopentane that had been cooled in liquid nitrogen. After mounting the strip in optimal cutting temperature (OCT) compound, muscle fibers were transversely sectioned with a cryostat (Reichert Jung Frigocut 2800 Cryostat, Reichert Microscope Services, Depew, NY, United States). For all histological and biochemical studies, alternate serial sections of the same muscle fibers were cut. These alternate sections were used for DIAm fiber type classification, measurement of fiber cross-sectional area, determination of fiber type proportions, calculation of the relative contribution of each fiber type to total DIAm volume, labeling mitochondria and measurement of mitochondrial morphology and volume density and measurement of SDH_*max*_.

### Immunohistochemical Determination of Muscle Fiber Types

The method for determining DIAm fiber types based on immunoreactivity to specific MyHC isoform antibodies has been previously described in detail (Sieck et al., 1995, 1996; Fogarty et al., 2019b). Briefly, three serial sections (cut at 10 μm) were fixed in 4% PFA for 10 min and then were blocked for 2 h in 10% bovine serum albumin. After rinsing with 0.1 PBS, primary antibodies for MyHC isoforms were applied and the sections were incubated overnight (4°C). One section was double-reacted with a primary antibody for MyHC_*slow*_ (Novus NBP2-50299) and MyHC_2A_ (Novus N1511) isoforms. A second section was reacted with primary antibodies for MyHC_2X_ (1:10 NPB1-22811) and the third was reacted for all-but-IIx MyHC (BF35) isoforms to identify fibers that co-express MyHC_2X_ and MyHC_2B_ isoforms (i.e., IIb fibers). A laminin primary antibody (anti-laminin Sigma L9393) was applied on all sections. The sections were thoroughly rinsed with 0.1 PBS and fluorescently conjugated secondary antibodies were applied at a 1:200 dilution for 2 h at room temperature. In one section, a secondary antibody, Alexa-Fluor 594 conjugated to IgG was used to visualize MyHC_*Slow*_ and another secondary antibody, Cy5 conjugated to IgM was used to visualize MyHC_2A_. In an adjacent section, a secondary antibody Alexa-Fluor 594 conjugated to IgG was used to visualize MyHC_2X_. In all sections, a secondary antibody Alexa-Fluor 405 conjugated to IgG was used to visualize laminin.

The fluorescent signals from the immunoreacted sections were imaged using a 20× oil-immersion objective (NA 1.0) on an Olympus FV2000 laser confocal microscope capable of simultaneous multicolor fluorescence imaging using an argon 405 nm, an argon 594 nm, and HeNe 543 nm lasers for imaging Alexa-Fluor 405, Alexa-Flour 594, and Cy5, respectively. Images were captured in a 1024 × 1024 pixel array, with similar acquisition parameters across preparations. Based on the fluorescence pattern, DIAm fibers were classified as type I, type IIa, and type IIx/IIb as outlined previously (Sieck et al., 1995, 1996; Fogarty et al., 2019b). It should be noted that in previous single fiber studies, we found that the MyHC_2B_ isoform was co-expressed in varying proportions with the MyHC_2X_ isoform (Geiger et al., 1999, 2000, 2001b). In addition, we were not able to validate an antibody for the MyHC_2B_ isoform. For these reasons, DIAm fibers displaying immunoreactivity for MyHC_2X_ were classified as type IIx/IIb. The proportion of different DIAm fiber types and their cross-sectional areas were determined using morphometric tools in ImageJ (United States National Institutes of Health, Bethesda, MD, United States,^1^ 1997–2018).

### Muscle Specific Force and Fatigue

Methods for measuring DIAm isometric specific force have been previously described in detail (Prakash et al., 1993a; Zhan et al., 1997). Briefly, the DIAm strip was suspended in a tissue bath, with the costal margin clamped and the central tendon tied with silk and attached to a force transducer (6350, Cambridge Technology, MA, United States), and optimal DIAm length (*L*o) and supramaximal stimulus settings were established. Electrical field stimulation was achieved *via* platinum plate electrodes placed on either side of the muscle, with stimulation current provided using a stimulator (701C, Aurora Scientific, ON, Canada). Supramaximal (∼150 mA) stimulus pulses (0.5 ms duration) were delivered at 5, 10, 20, 30, 40, 50, 75, and 100 Hz in 1 s trains.

Fatigue of the DIAm was assessed using a pattern of direct muscle stimulation as previously described (Sieck et al., 1989b, 1991; Lewis et al., 1992; Fogarty et al., 2019b). Briefly, supramaximal (∼150 mA) stimulus pulses (0.05 ms duration) were delivered at 40 Hz in 333 ms trains repeated each second [33% duty cycle, approximating the duty cycle of ventilation (Sieck et al., 1984; Richards et al., 2018)] for 120 s.

Output of the force transducer data was digitized (1 kHz sampling rate) and recorded in LabChart software (ADInstuments, Dunedin, New Zealand). Specific force of the DIAm was calculated by normalizing force to the estimated cross-sectional area of the DIAm strip {muscle cross-sectional area = muscle strip weight (g)/[*L*o (cm) × 1.056 g/cm^3^]} and expressed as N/cm^2^.

### Modeling Changes in Specific Force

To model the relative contribution of different fiber types to DIAm force generation in 6- and 24-month rats, we used previously published data for maximum specific force generated by different fiber types that were obtained using single permeabilized fibers maximally activated at a pCa = 4.0 (Geiger et al., 1999, 2000, 2001b). The model assumed the previously reported ∼15% decrease in total MyHC isoform concentration with aging (Elliott et al., 2016) were exclusive to the vulnerable type IIx/IIb fibers, as previously established in male and female Fischer 344 rats (Khurram et al., 2018a; Fogarty et al., 2019a,b, 2020). The specific DIAm force in the model was obtained by multiplying the fiber type-specific contribution to total DIAm volume by the calculated fiber type specific specific force, and normalizing for interstitial space. The parameters used in modeling DIAm specific force are reported in Table 1. For modeling the specific force following 2 min of repeated fatiguing activations, we assumed type-specific fatigue indices (0.8 for type I and IIa and 0.1 for type IIx/IIb), based on prior reports (Fournier and Sieck, 1988).

**TABLE 1:** Single fiber characteristics for DIAm Force Model.

Fiber type	Fatigue index	Contribution to total DIAm volume (%) 6-month	Maximum specific force (N/cm^2^) 6-month	Residual specific force (N/cm^2^) 6-month	Contribution to total DIAm volume (%) 24-month	Maximum specific force (N/cm^2^) 24-month	Residual specific force (N/cm^2^) 24-month
Type I	0.8^*a*^	14	20^*b*^	16^*d*^	28	20	16^*d*^
Type IIa	0.8^*a*^	14	24^*b*^	19^*d*^	21	24	19^*d*^
Type IIx/IIb	0.1^*a*^	72	33^*b*^	3^*d*^	51	23^*c*^	2^*d*^

*^*a*^Fatigue index based on past reports for DIAm motor units (Fournier and Sieck, 1988).*

*^*b*^Specific force data from DIAm single fibers was published previously (Geiger et al., 2000, 2001a, 2002).*

*^*c*^Derived from observed reductions in total MyHC expression, and attributed to the vulnerable type IIx/IIb fibers (Elliott et al., 2016).*

*^*d*^Residual specific forces following fatigue calculated from values derived from a, b, and c.*

### Mitochondrial Volume and Morphology

The methods used to label and image mitochondria using MitoTracker Green and confocal microscopy have been previously described in detail (Delmotte et al., 2017, 2020). Briefly, 10 μm transverse sections of the same muscle fibers were incubated for 30 min in a MitoTracker Green solution (1.5 μL MitoTracker Green in 5 mL PBS). After thoroughly washing the sections with PBS three times (10 min each), the sections were cover-slipped for imaging. Mitochondria were visualized using an Olympus FV2000 laser scanning confocal microscope (Olympus Life Sciences Solutions, Waltham, MA, United States) at 16-bit resolution (1.096 gray levels), 1024 × 1024 pixels using a 40× Plan Apo oil-immersion objective (NA 1.40). The confocal imaging techniques used have been previously reported in detail (Prakash et al., 1993b; Sieck et al., 1999). The calculated point spread function for the 40× objective was used to set a *Z*-axis step size of 0.5 μm (voxel dimensions: 0.207 × 0.207 × 0.5 μm). The photomultiplier settings and laser intensity were adjusted using two different regions of interests (ROI): one with no MitoTracker Green signal and a second ROI with saturated MitoTracker Green signal. The ROI with no MitoTracker Green signal was used to adjust the black level so that the gray levels were not saturated, but the gray levels were <10% of the dynamic range. The other ROI with the most intense MitoTracker Green fluorescence was used to adjust the gain to maximize the dynamic range, while preventing saturation of the image.

To improve spatial resolution, each 0.5 μm optical slice within the 10 μm *Z*-stack (20 optical slices) was deconvolved using a blind deconvolution algorithm (Point Scan Confocal, three iterations; NIS-Elements; Nikon Instruments Inc., SCR_014329) (Fogarty et al., 2021b). This deconvolution algorithm increases spatial resolution twofold. The deconvolved images were then processed for background correction, ridge filter detection, skeletonization, and thresholding using ImageJ software (see text footnote 1, Fiji, SCR_002285), as previously described (Koopman et al., 2005, 2006; Delmotte et al., 2017). In this method, the gray level of MitoTracker Green fluorescence in the images was thresholded to identify MitoTracker containing voxels, and binary images were created (Figure 3). These binarized images were reconstructed in 3-D using ImageJ and NIS-Elements.

The ImageJ/Fiji Mitochondria Analyzer plug-in was used to assess mitochondrial morphology, including mitochondrial volume, surface area, mean branch length (μm), and mitochondrial complexity index (MCI) (Vincent et al., 2019). MCI is calculated using the following equation:


$$M\,C\,I=\frac{S\,A^{3}}{16\,\pi^{2}\,V^{2}}$$

where *SA* is total mitochondrial surface area within a fiber. *V* is the total mitochondrial volume within the muscle. Mitochondria that are more fragmented exhibit a lower mean branch length and a lower MCI.

### Maximum Velocity of Succinate Dehydrogenase Reaction

The quantitative histochemical procedure for measuring SDH_*max*_ in muscle fibers has been previously described in detail (Sieck et al., 1986; Blanco et al., 1988). Briefly, 6 μm thick transverse serial sections of the same DIAm fibers were placed in a solution containing 80 mM succinate with 1.5 mM nitro blue tetrazolium (NBT – reaction indicator), 5 mM EDTA, 0.2 mM mPMS, and 0.1 mM azide in 0.1 M phosphate buffer (pH = 7.6) kept at 25°C. Previously, we confirmed that succinate concentrations >80 mM does not substrate limit the SDH reaction in DIAm fibers. In the quantitative histochemical procedure, the progressive precipitation of a reduced NBT (NBT diformazan, NBT_*dfz*_) is used as the reaction indicator. The accumulation of the NBT_*dfz*_ precipitate in muscle fibers was quantified using a computer-based imaging system consisting of a light microscope (Olympus IX71, Olympus America, Melville, NY, United States) with an attached camera (Hamamatsu ORCA Flash 4.0, Model C11440). An interference filter (570 nm) was used to limit the spectral range of the light source to the maximum absorption wavelength of NBT_*dfz*_. Images were acquired every 15 s as the SDH reaction proceeded using a 60× objective (1.0 NA) and captured in a 1024 × 1024 pixel array, with similar acquisition parameters across preparations. In a separate preliminary study, we confirmed that the OD of fibers exposed to a control solution containing no succinate substrate was the same as that for fibers exposed to the succinate-containing solution at the initiation of the reaction (i.e., at time 0 s). Thus, [NBT_*dfz*_] (OD at 570 nm) in delineated DIAm fibers was measured every 15 s, and the linearity of the change in [NBT_*dfz*_] during the SDH reaction was confirmed for all fibers. From repeated images, the SDH_*max*_ was calculated using the Beer–Lambert–Bouguer law (below):


$$\frac{d\,\left[N\,B\,T_{d\,f\,z}\right]}{d\,t}=\frac{d\,O\,D/d\,t}{k\,l}$$

where *OD* is the average optical density within the boundary of a DIAm fiber, *k* is the molar extinction coefficient for NBT_*dfz*_ (26,478 mol/cm), and *l* is the path length of light absorbance (6 μm). *OD* was calibrated with a series of known gray levels at 16-bit resolution (1096 gray levels).

In separate studies, we assessed whether the measurement of SDH_*max*_ reflects the maximum respiratory capacity of muscle fibers by using a stress test similar to that used in respirometry systems. In the stress test, the muscle sections were treated (or untreated) with 1 mM FCCP (2-[2-[4-(trifluoromethoxy)phenyl]hydrazinylidene]-propanedinitrile), a protonophore that uncouples O_2_ consumption from the proton gradient. In a second part of the stress test, alternate sections of the same fibers were treated (or untreated) with rotenone (1 mM) to inhibit complex I of the electron transport chain and antimycin A (1 mM) to inhibit complex III (Ly and Ryall, 2017; Jacques et al., 2020). Respirometry systems rely on changes in O_2_ consumption rate in response to these same compounds to determine the basal and maximum respiratory capacity (O_2_ consumption rate). The rate of the SDH reaction was unaffected by treatment with FCCP across all fiber types, indicating that SDH_*max*_ reflects the maximum respiratory capacity of muscle fibers. In contrast, the rate of the SDH reaction was markedly blunted by treatment with rotenone and antimycin A across all DIAm fiber types (*P* < 0.002 for all combinations, Bonferroni *post hoc* tests), again consistent with SDH_*max*_ as a measure of the maximum respiratory capacity of muscle fibers.

Based on the Beer–Lambert–Bouguer law, SDH_*max*_ within individual DIAm fiber volumes was expressed as millimoles of fumarate/L_*fiber*_/min. The SDH_*max*_ per fiber volume was also normalized for mitochondrial volume density within DIAm fibers to calculate SDH_*max*_ per mitochondrial volume, which was expressed as millimoles of fumarate/L_*mito*_/min. This provided a measure of the maximum respiratory capacity of mitochondria within type identified DIAm fibers.

### Statistical Methods

The number of animals required for adequate power when assessing force and fatigue data was estimated based on previous data (*n* = 8–10 per group) (Gosselin et al., 1994; Fogarty et al., 2019b, 2020). Power analysis to determine the sample size for the SDH procedures (*n* = 15 fibers per type per animal) based on previous SDH_*max*_ data from type-identified DIAm fibers in 6-month adult rats (Sieck et al., 1995; Fogarty et al., 2020). The expected effect size (Cohen’s *d*) was calculated with an *a priori* biologically relevant difference of 25% and equal variance, with sample size required estimated using *d* = 1.4, α = 0.05, and β = 0.8. Two-way repeated measures ANOVA and Bonferroni *post hoc* tests were used to compare differences between age groups and factor (fiber type). Although we were not powered to detect a sex difference, and our force, fatigue, and DIAm fiber type specific outcome measurements have previously included both males and females, sex differences were explored using either two- or one-way ANOVAS. outcome Linear relations were assessed with Pearson’s coefficients. All data were assessed for normality with Shapiro–Wilk tests. Significance was set as *P* < 0.05, all data are presented as mean ± 95% confidence intervals (CIs), unless otherwise stated. All statistics were performed in Prism 8 (GraphPad, CA, United States).

## Results

### Fiber-Type Proportions, Cross-Sectional Areas, and Contributions to Total Diaphragm Muscle Mass

**FIGURE 1:**
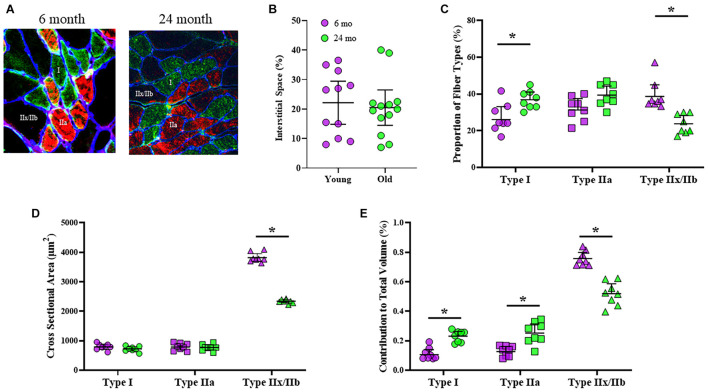
**(A)** Representative, triple-labeled images of fiber typing based on immunoreactivity to primary antibodies for MyHC_*slow*_ (red) and MyHC_2A_ (green) isoforms with the inclusion of a primary antibody for laminin (blue) to clearly define the muscle fiber borders. **(B)** Scatterplot showing that the relative contribution of interstitial space to total DIAm cross-sectional area was unchanged between 6-month (purple) and 24-month (green) Fischer 344 rats (Student’s unpaired *t*-test, *P* > 0.05). **(C)** Scatterplot showing an increased proportion of type I fibers (circles) and a decreased proportion of type IIx/IIb fibers (triangles) in 24-month compared with 6-month rats (two-way ANOVA, *P < 0.05). **(D)** Scatterplot showing the mean fiber cross-sectional areas of type I (circles), type IIa (squares), and type IIx/IIb (triangles) fibers. Note the selective reduction in cross-sectional area of type IIx/IIb DIAm fibers in 24-month compared with 6-month rats (two-way ANOVA, *P < 0.05). **(E)** Scatterplot showing the relative contributions of each fiber type to total DIAm volume. Note the increased contribution of both type I and IIa DIAm fibers and the reduction in type IIx/IIb fibers to total DIAm volume in 24-month compared with 6-month rats (two-way ANOVA, *P < 0.05). All data sets include female and male rats per age group (see Table 2) with 15 fibers per type in each DIAm.

Classification of DIAm fiber types was based on immunoreactivity to specific MyHC antibodies (Figure 1A). The proportional volume of DIAm occupied by interstitial, non-muscle fiber space was unchanged in 6-month (22 ± 7%) compared with 24-month (20 ± 6%) rats (*P* = 0.78, unpaired *t*-test; Figure 1B), similar to past reports (Khurram et al., 2018b). The proportions of different fiber types in the DIAm were dependent on the interaction of fiber type and age [*F*_(2,44)_ = 13.8, *P* < 0.0001], but not fiber type alone [*F*_(1.9,41.2)_ = 2.4, *P* = 0.10], nor age alone [*F*_(1,22)_ = 1.2, *P* = 0.28; two-way ANOVAs; Figure 1C]. Bonferroni *post hoc* tests showed an increase in the proportion of type I fibers in 24-month compared with 6-month (*P* = 0.01) rats, and a decrease in the proportion of type IIx/IIb fibers in 24-month compared with 6-month rats (*P* = 0.002; Figure 1C). The cross-sectional areas of DIAm fibers were dependent on fiber type [*F*_(1.2,25.9)_ = 824.9, *P* < 0.0001], age [*F*_(1,22)_ = 43.0, *P* < 0.0001], and the interaction between fiber type and age [*F*_(2,44)_ = 68.5, *P* < 0.0001; two-way ANOVAs; Figure 1D]. Bonferroni *post hoc* test show no difference in the cross sectional areas of type I (6-month: 802 ± 105 μm^2^; 24-month: 734 ± 74 μm^2^; *P* = 0.46) nor type IIa fibers (6-month: 755 ± 87 μm^2^; 24-month: 793 ± 92 μm^2^; *P* > 0.99) between 6-month and 24-month rats (Figure 1D). However, the cross-sectional areas of type IIx/IIb DIAm fibers were ∼39% smaller in 24-month (2306 ± 249 μm^2^) compared to 6-month (3584 ± 437 μm^2^) rats (*P* < 0.0001; Figure 1D). The relative contributions of different fiber types to total DIAm volume was dependent on fiber type [*F*_(1.6,34.2)_ = 268.2, *P* < 0.0001] and the interaction between fiber type and age [*F*_(2,44)_ = 36.3, *P* < 0.0001], but not age alone [*F*_(1,22)_ = 0.4, *P* = 0.57; two-way ANOVAs; Figure 1E]. Bonferroni *post hoc* test showed a greater relative contribution of type I (*P* = 0.002) and type IIa (*P* < 0.0001) fibers to total DIAm volume in 24-month compared with 6-month rats, and a lower relative contribution of type IIx/IIb fibers to total DIAm volume in 24-month compared with 6-month rats (*P* < 0.0001; Figure 1E).

When type specific cross-sectional areas of DIAm fibers were stratified by sex, we observed no significant differences between males and females [*F*_(1,20)_ = 0.3, *P* = 0.56; three-way ANOVA; Table 2], with Bonferroni *post hoc* tests confirming a selective effect of reduced cross-sectional area of type IIx/IIb fibers in 24-month female (*P* = 0.02) and male (*P* = 0.04) rats (Table 2).

**TABLE 2:** Results for DIAm cross-sectional area, force, and fatigue stratified by sex.

Parameter	6-months old (*n*)		24-months old (*n*)	ANOVA
DIAm fiber cross-sectional area (μm^2^)	Type I	♀: 812 ± 111 (*6*) ♂: 792 ± 145 (5)	♀: 743 ± 75 (*6*) ♂: 726 ± 156 (*7*)	Fiber type: *F* = 867.7, *P* < 0.0001 Age: *F* = 42.5, *P* < 0.0001
	Type IIa	♀: 780 ± 166 (*6*) ♂: 725 ± 149 (*5*)	♀: 785 ± 127 (*6*) ♂: 802 ± 125 (*7*)	Sex: *F* = 0.3, *P* = 0.57 Fiber type × age: *F* = 74.5, *P* < 0.0001
	Type IIx/IIb	♀: 3418 ± 573 (*6*) ♂: 3784 ± 366 (*5*)	♀: 2334 ± 71 (*6*) ♂: 2282 ± 151 (*7*)	Fiber type × sex: *F* = 1.3, *P* = 0.26 Age × sex: *F* = 0.7, *P* = 0.41 Fiber type × age × sex: *F* = 2.4, *P* = 0.11
Twitch specific force (N/cm^2^)	♀: 6.2 ± 2.3 (5) ♂: 6.3 ± 1.3 (*5*)		♀: 4.5 ± 0.8 (5) ♂: 6.1 ± 1.1 (*5*)	Age: *F* = 3.1, *P* = 0.10 Sex: *F* = 2.5, *P* = 0.13 Age × sex: *F* = 2.2, *P* = 0.15
Maximum specific force (N/cm^2^)	♀: 22.6 ± 4.3 (5) ♂: 24.2 ± 7.5 (*5*)		♀: 15.7 ± 1.9 (5) ♂: 17.3 ± 2.0 (*5*)	Age: *F* = 18.3, *P* = 0.0006 Sex: *F* = 1.0, *P* = 0.34 Age × sex: *F* < 0.1, *P* = 0.98
Fatigue index	♀: 23.3 ± 10.1 (5) ♂: 22.6 ± 4.0 (*5*)		♀: 33.9 ± 4.4 (5) ♂: 33.8 ± 9.2 (*5*)	Age: *F* = 16.5, *P* = 0.0009 Sex: *F* < 0.1, *P* = 0.89 Age × sex: *F* < 0.1, *P* = 0.99
Residual specific force following fatigue (N/cm^2^)	♀: 4.1 ± 2.2 (5) ♂: 4.3 ± 0.9 (*5*)		♀: 4.6 ± 1.5 (5) ♂: 4.7 ± 1.6 (*5*)	Age: *F* = 0.6, *P* = 0.43 Sex: *F* < 0.1, *P* = 0.83 Age × sex: *F* < 0.1, *P* = 0.95

*For DIAm fiber cross-sectional area, results derive from three-way ANOVA, for all other assessments, two-way ANOVAs. Note no observed effect of sex on any parameter assessed here. All summary data, mean ± 95% confidence interval, with n indicated in bracketed italics.*

### Age-Related Changes in Maximum Diaphragm Muscle Specific Force

**FIGURE 2:**
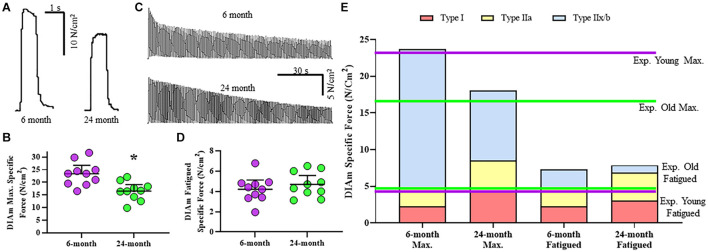
**(A)** Representative raw tracings of the maximum specific force generated at 75 Hz stimulation by the DIAm from a 6-month compared with a 24-month Fischer 344 rat (both male rats). **(B)** Scatterplot showing reduced DIAm maximum specific force in 24-month compared with 6-month rats (Student’s unpaired *t*-test, *P < 0.05). **(C)** Representative traces of the force decline (fatigue) of the DIAm during repeated stimulation at 40 Hz in 0.33 s duration trains repeated each second across a 2 min period from a 6-month compared with a 24-month rat. **(D)** Scatterplot showing unchanged DIAm specific force following 2 min of repeated activations in 24-month compared with 6-month rats (Student’s unpaired *t*-test, *P* > 0.05). **(E)** The maximum specific force contributed by type I (red boxes), type IIa (yellow boxes), and type IIx/IIb (blue boxes) DIAm fibers was modeled for 6-month compared with 24-month rats based on differences in fiber type contributions to total DIAm volume and previously reported differences in specific force generated by each fiber type (see Table 1). Experimental observations of DIAm specific force were superimposed in dashed lines for 6-month (dashed purple line) and 24-month (dashed green line) rats.

Specific isometric force generation was assessed in 6- and 24-month rats (Figure 2A). No significant change in DIAm specific twitch force was observed between 6-month (5.9 ± 1.0 N/cm^2^, *n* = 10) and 24-month (4.6 ± 1.1 N/cm^2^, *n* = 10) rats (*P* = 0.08, unpaired *t*-test). When stratified for sex, we observed no differences in twitch force between females and males [*F*_(1,16)_ = 2.5, *P* = 0.13; two-way ANOVA; Table 2], nor with age [*F*_(1,16)_ = 3.1, *P* = 0.10; two-way ANOVA; Table 2].

**FIGURE 3:**
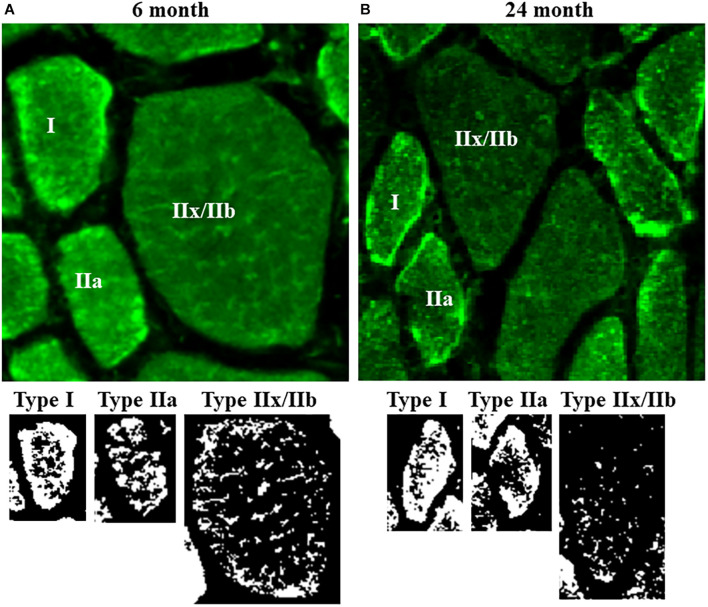
**(A)** Representative photomicrographs of mitochondria labeled using MitoTracker Green in DIAm fibers from 6-month and 24-month Fischer 344 rats in alternate serial sections (same fibers used for MyHC fiber type classification and SDH_*max*_ measurements) cut at 10 μm thickness. MitoTracker Green fluorescence was visualized using an Olympus FV2000 laser scanning confocal microscope. **(B)** Fluorescence intensity was thresholded to produce binary images of the MitoTracker Green labeled mitochondria.

The maximum DIAm specific force (generated at 75 Hz stimulation) was reduced by 29% in 24-month (16.5 ± 2.6 N/cm^2^, *n* = 10) compared with 6-month rats (23.4 ± 3.4 N/cm^2^, *n* = 10; *P* = 0.002, unpaired *t*-test; Figure 2B). When stratified for sex, we observed no differences in maximum DIAm force between females and males [*F*_(1,16)_ = 1.0, *P* = 0.34; two-way ANOVA; Table 2], however, the effect of reduced force with age [*F*_(1,16)_ = 18.3, *P* = 0.0006; two-way ANOVA; Table 2] was maintained in both 24-month female (*P* = 0.01, Bonferroni *post hoc* test) and male (*P* = 0.04, Bonferroni *post hoc* test) rats.

Age-related changes in maximum specific force in DIAm from 6- and 24-month rats were estimated in a model based on measured changes in fiber type proportions, cross-sectional areas, the relative contribution of each fiber type to total DIAm volume. The model also included previously reported differences in maximum specific force across fiber types (Geiger et al., 1999, 2000, 2001b). The model also accounted for an ∼37% combined reduction in the concentration of MyHC_2X_ and/or MyHC_2B_ isoforms previously reported (Elliott et al., 2016) and the consequent impact on the specific force of type IIx/IIb DIAm fibers. The estimates of maximum DIAm specific force were compared to actual measured specific force for 6- and 24-month rats (Figure 2E). Notably, in 6-month rats, the estimated maximum specific force closely approximated the maximum specific force that was observed experimentally (∼2.5% difference; Figure 2E). For 24-month rats, the estimate of maximum DIAm specific force predicted by modeling also closely approximated that observed experimentally (Figure 2E).

### Residual Diaphragm Muscle Specific Force Following Fatigue

Diaphragm muscle force generated at 40 Hz stimulation declined with repetitive activation across a 2-min period in both 6- and 24-month rats, reflecting force fatigue (Figure 2C). Due to the higher initial force generated by the DIAm in 6-month rats, the decline in force was greater in 6-month compared with 24-month rats. This decline in force was reflected by the fatigue index calculated as the ratio of residual force after 2 min to the initial force. Accordingly, the fatigue index of the DIAm of 24-month rats was improved compared with 6-month rats (6-month: 22.9 ± 4.2%, *n* = 10; 24-month: 33.8 ± 4.0%, *n* = 10; *P* = 0.0004, unpaired *t*-test). When stratified for sex, we observed no differences in fatigue index between females and males [*F*_(1,16)_ < 0.1, *P* = 0.89; two-way ANOVA; Table 2].

This apparent improvement in fatigue resistance was due primarily to the lower initial specific force generated by the DIAm from the 24- compared with 6-month rats. Importantly, the residual specific forces of the DIAm following 2 min of repetitive activation were not statistically different between 6- (4.2 ± 0.9 N/cm^2^, *n* = 10) and 24-month (4.7 ± 1.1 N/cm^2^, *n* = 10) rats (*P* = 0.41, unpaired *t*-test; Figure 2D). When stratified for sex, we observed no differences in residual DIAm force following fatigue between females and males [*F*_(1,16)_ < 0.1, *P* = 0.83; two-way ANOVA; Table 2].

Similar to the maximum specific force, we modeled the residual DIAm specific force predicted after 2 min of repetitive activation in 6- and 24-month rats (Figure 2E). Our model accounted for differences in fatigue indices that define motor unit types (Fournier and Sieck, 1988), fiber type specific contribution to total DIAm volume and fiber-type dependent specific forces (Geiger et al., 1999, 2000, 2001a,b). In contrast to the model of maximum specific force, the model of residual force following fatigue did not match the experimentally observed results for the DIAm in both 6- and 24-month rats (Figure 2E). The actual residual force measured experimentally was ∼27–30% less than that predicted by our model (∼4.7 N/cm^2^ predicted vs. ∼6.5 N/cm^2^ actual; Figure 2E).

### Mitochondrial Volume

**FIGURE 4:**
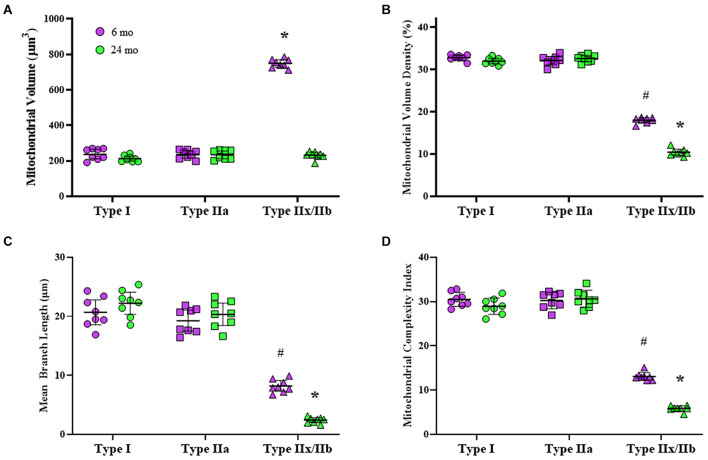
**(A)** Scatterplot showing that mean mitochondrial volume in type I (circles) and IIa (squares) DIAm fibers is unchanged between 6-month (purple) and 24-month (green) Fischer 344 rats. Mitochondrial volume in type IIx/IIb (triangles) DIAm fibers was significantly lower in 24-month compared with 6-month rats (two-way ANOVA, *P < 0.05). **(B)** Scatterplot of the mean mitochondrial volume density of type I, type IIa, and type IIx/IIb fibers. There were no differences in mitochondrial volume densities between type I and IIa fibers at either age. Mitochondrial volume density in type IIx/IIb fibers was lower than that of type I or IIa fibers at both ages and was significantly reduced in 24-month compared with 6-month rats (two-way ANOVA, *P < 0.05). **(C)** Scatterplot showing that mean mitochondrial branch length (μm) in type I and IIa fibers was similar at both ages and was not different between 6-month and 24-month rats. Mitochondrial branch length in type IIx/IIb fibers was shorter than that in type I and IIa fibers at both ages. In addition, mitochondrial branch length in type IIx/IIb fibers was reduced in 24-month compared with the 6-month rats (two-way ANOVA, *P < 0.05). **(D)** Scatterplot showing mean DIAm fiber mitochondrial complexity index (MCI). The MCI was similar between type I and IIa fibers and did not change with age. The MCI of in type IIx/IIb DIAm fibers was lower than that of type I and IIa fibers at both ages. In addition, the MCI in type IIx/IIb DIAm fibers was reduced in 24-month compared with the 6-month rats (two-way ANOVA, *P < 0.05). # indicated difference between type IIx/IIb fibers and type I and IIa fibers of all ages.

Mitochondria within type identified DIAm fibers were labeled using MitoTracker Green, imaged using confocal microscopy and converted to binary images post acquisition (Figure 3). Within 10 μm transverse sections of DIAm fibers, mitochondrial volume was dependent on age [*F*_(1,42)_ = 658.9, *P* < 0.0001], fiber type [*F*_(2,42)_ = 598.3, *P* < 0.0001], and the interaction between age and fiber type [*F*_(2,42)_ = 561.3, *P* < 0.0001; two-way ANOVA; Figure 4A]. Bonferroni *post hoc* tests showed no age-associated differences in mitochondrial volume in type I (6-month: 238 ± 27 μm^3^; 24-month: 212 ± 14 μm^3^; *P* = 0.64) and IIa (6-month: 237 ± 20 μm^3^; 24-month: 234 ± 22 μm^3^; *P* > 0.99) fibers (Figure 4A). However, mitochondrial volume was markedly reduced with age in type IIx/IIb fibers (6-month: 749 ± 21 μm^3^; 24-month: 231 ± 17 μm^3^; *P* < 0.0001; Figure 4A). Mitochondrial volume was greater in type IIx/IIb fibers from 6-month rats compared with all other fibers of all ages (*P* < 0.05 for all comparisons; Figure 4A). However, the ∼3.8-fold greater mitochondrial volume in type IIx/IIb DIAm fibers in 6-month rats was disproportionately less than the ∼4-5-fold difference in fiber volume, as reflected in differences in mitochondrial volume density across fiber types (Figure 4A). Accordingly, mitochondrial volume density was significantly lower in type IIx/IIb DIAm fibers compared with type I and IIa fibers in both 6- and 24-month rats (*P* < 0.05 for all comparisons; Figure 4B). Mitochondrial volume density was dependent on age [*F*_(1,42)_ = 115.5, *P* < 0.0001], fiber type [*F*_(2,42)_ = 2397, *P* < 0.0001], and the interaction between age and fiber type [*F*_(2,42)_ = 101.7, *P* < 0.0001; two-way ANOVA; Figure 4B]. Bonferroni *post hoc* tests showed no age-associated differences in mitochondrial volume density in type I (6-month: 32.8 ± 0.5; 24-month: 32.0 ± 0.6; *P* = 0.85) and IIa (6-month: 32.2 ± 1.0; 24-month: 32.6 ± 0.7; *P* > 0.99) fibers (Figure 4B). However, mitochondrial volume density was markedly reduced with age in type IIx/IIb fibers (6-month: 18.0 ± 0.6; 24-month: 10.4 ± 0.7; *P* < 0.0001; Figure 4B).

### Mitochondrial Morphology

The morphology of mitochondria within DIAm fibers differed across fiber types. The mean mitochondrial branch length was different across fiber types [*F*_(2.42)_ = 335.1, *P* < 0.0001] and affected by the interaction between fiber type and age [*F*_(2.42)_ = 17.9, *P* < 0.0001], although not by aging alone [*F*_(1.42)_ = 3.7, *P* = 0.07; two-way ANOVA; Figure 4C]. Bonferroni *post hoc* tests showed no age-related difference in mean mitochondrial branch lengths in type I (6-month: 20.7 ± 2.1 μm; 24-month: 22.2 ± 1.9 μm; *P* > 0.99) and type IIa (6-month: 19.3 ± 1.7 μm; 24-month: 20.3 ± 1.9 μm; *P* > 0.99) DIAm fibers (Figure 4C). However, the mean mitochondrial branch length in type IIx/IIb fibers was lower in both 6- (8.2 ± 0.9 μm) and 24-month rats (2.4 ± 0.4 μm) compared with type I and IIa fibers (*P* < 0.0001; Figure 4C), reflecting mitochondrial fragmentation. In addition, mean mitochondrial branch length in type IIx/IIb DIAm fibers from 24-month rats were significantly lower than that of type IIx/IIb fibers from 6-month animals (*P* < 0.0001; Figure 4C).

The MCI also varied across DIAm fiber types being lowest in type IIx/IIb fibers in both 6- and 24-month rats, reflecting greater mitochondrial fragmentation (Figure 4D). The MCI in DIAm fibers was affected by age [*F*_(1.42)_ = 39.4, *P* < 0.0001], fiber type [*F*_(2.42)_ = 950.3, *P* < 0.0001], and the interaction between age and fiber type [*F*_(2.42)_ = 26.3, *P* < 0.0001; two-way ANOVA; Figure 4D]. Bonferroni *post hoc* tests show unchanged MCI with age in type I (6-month: 30.5 ± 1.3; 24-month: 28.9 ± 0.6; *P* > 0.99) and type IIa (6-month: 30.2 ± 1.6; 24-month: 30.6 ± 1.6; *P* > 0.99) DIAm fibers (Figure 4D). We did observe a reduction in the MCI in type IIx/IIb fibers of 24-month rats (5.9 ± 0.5) compared with 6-month (13.1 ± 0.7; *P* < 0.0001; Figure 4D).

### Maximum Velocity of the Succinate Dehydrogenase Reaction per Muscle Fiber Volume

**FIGURE 5:**
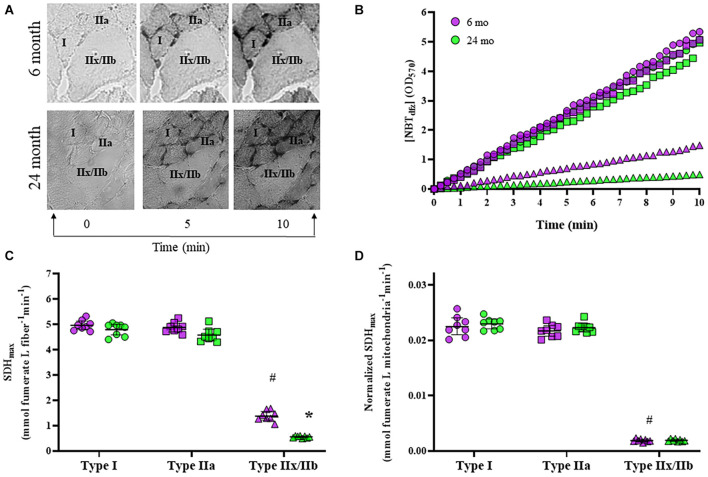
**(A)** Representative images of the accumulation of NBT_*dfz*_ (SDH reaction indicator) in DIAm fibers from 6- and 24-month Fischer 344 rats at 0, 5, and 10 min during the progression of the SDH reaction. **(B)** Measurements of mean fiber OD [indicating [NBT_*dfz*_] were obtained every 15 s during the SDH reaction for type I (circles), IIa (squares), and IIx/IIb (triangles) DIAm fibers from both 6-month (purple) and 24-month (green) male and female rats]. The SDH reaction was found to be highly linear (*R*^2^ = 0.99) during this period. From the slope of accumulation of [NBTdfz] (*d*OD/*dt*), the maximum velocity of the SDH reaction (SDH_*max*_) was determined for each fiber. **(C)** SDH_*max*_ normalized to fiber volume was higher in type I and IIa DIAm fibers compared to type IIx/IIb fibers. SDH_*max*_ normalized to fiber volume was not different in type I and IIa DIAm fibers between 6-month and 24-month rats. SDH_*max*_ normalized to fiber volume in type IIx/IIb fibers was lower than that in type IIx/IIb DIAm fibers in 24-month compared with 6-month rats (two-way ANOVA, *P < 0.05). **(D)** SDH_*max*_ normalized to mitochondrial volume per fiber was higher in type I and IIa DIAm fibers compared to type IIx/IIb fibers. SDH_*max*_ normalized to mitochondrial volume per fiber was not different in type I and IIa DIAm fibers between 6-month and 24-month rats. SDH_*max*_ normalized to mitochondrial volume in type IIx/IIb DIAm fibers was lower in 24-month compared with 6-month rats (two-way ANOVA, *P < 0.05). # indicated difference between type IIx/IIb fibers and type I and IIa fibers of all ages.

We measured the SDH reaction across a 10-min period in type I, type IIa, and type IIx/IIb DIAm fibers in 6- and 24-month rats, and found that changes in [NBT_*dfz*_] (average OD) within DIAm fibers was highly linear in each case (Figures 5A,B). We found that SDH_*max*_ was dependent on age [*F*_(1,42)_ = 51.6, *P* < 0.0001], fiber type [*F*_(2,42)_ = 1787, *P* < 0.0001], and the interaction between age and fiber type [*F*_(2,42)_ = 11.2, *P* = 0.0001; two-way ANOVA; Figure 5C]. Bonferroni *post hoc* tests showed no age-associated differences in SDH_*max*_ in type I (6-month: 4.97 ± 0.17 mmol fumarate L fiber^–1^ min^–1^; 24-month: 4.80 ± 0.21 mmol fumarate L fiber^–1^ min^–1^; *P* > 0.99) and IIa (6-month: 4.88 ± 0.17 mmol fumarate L fiber^–1^ min^–1^; 24-month: 4.58 ± 0.22 mmol fumarate L fiber^–1^ min^–1^; *P* = 0.11) DIAm fibers (Figure 5C). However, SDH_*max*_ per fiber volume was markedly reduced with age in type IIx/IIb DIAm fibers (6-month: 1.39 ± 0.17 mmol fumarate L fiber^–1^ min^–1^; 24-month: 0.56 ± 0.03 mmol fumarate L fiber^–1^ min^–1^; *P* < 0.0001; Figure 5C).

### Maximum Velocity of the Succinate Dehydrogenase Reaction per Mitochondrial Volume

The SDH_*max*_ per fiber volume was normalized for mitochondrial volume density to determine SDH_*max*_ per mitochondrial volume. We found that SDH_*max*_ per mitochondrial volume was dependent on fiber type [*F*_(2,42)_ = 1943, *P* < 0.0001] but not age [*F*_(1,42)_ = 1.2, *P* = 0.28], and the interaction between age and fiber type [*F*_(2,42)_ = 0.3, *P* = 0.76; two-way ANOVA; Figure 5D]. Bonferroni *post hoc* tests showed no age-associated differences in SDH_*max*_ per mitochondrial volume in type I (6-month: 0.023 ± 0.002 mmol fumarate L mitochondria^–1^ min^–1^; 24-month: 0.023 ± 0.001 mmol fumarate L mitochondria^–1^ min^–1^; *P* > 0.99), IIa (6-month: 0.022 ± 0.002 mmol fumarate L mitochondria^–1^ min^–1^; 24-month: 0.022 ± 0.001 mmol fumarate L mitochondria^–1^ min^–1^; *P* > 0.99), or IIx/IIb fibers (6-month: 0.0019 ± 0.0003 mmol fumarate L mitochondria^–1^ min^–1^; 24-month: 0.0019 ± 0.0002 mmol fumarate L mitochondria^–1^ min^–1^; *P* > 0.99; Figure 5C). However, SDH_*max*_ per mitochondrial volume was markedly reduced with in type IIx/IIb fibers compared with type I and IIa fibers of all ages (*P* < 0.05 for all comparisons; Figure 5D).

### Relation Between Maximum Velocity of the Succinate Dehydrogenase Reaction per Fiber Volume and Mitochondrial Volume Density

**FIGURE 6:**
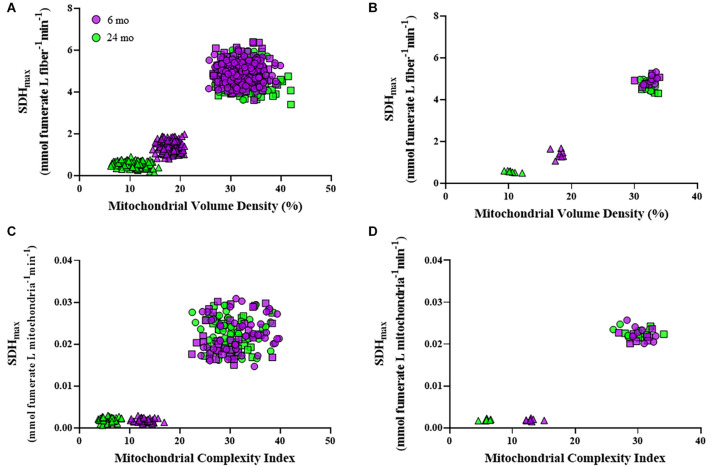
Scatter plots showing the relationships between SDH_*max*_ normalized to fiber volume and mitochondrial volume density for all type I (circles), IIa (squares), and IIx/IIb (triangles) DIAm fibers **(A)** and for the mean values per fiber type for each Fischer 344 rat **(B)**. Note the overlapping clusters for values from type I and IIa DIAm fibers, with a clear separation from values for type IIx/IIb fibers at both ages. In type IIx/IIb fibers, there was a clear separation of values for both SDH_*max*_ normalized to fiber volume and mitochondrial volume density in 6-month compared with 24-month rats. Scatter plots showing the relationships between SDH_*max*_ normalized to mitochondrial volume per fiber and mitochondrial complexity index (MCI) for all type I (circles), IIa (squares), and IIx/IIb (triangles) DIAm fibers **(C)** and for the mean values per fiber type for each animal **(D)**. Note the overlapping clusters for values from type I and IIa DIAm fibers, with a clear separation from values for type IIx/IIb fibers at both ages. In type IIx/IIb fibers, there was a clear separation of values for both SDH_*max*_ normalized to mitochondrial volume per fiber and MCI in 6-month compared with 24-month rats.

Within an individual DIAm fiber, the relation between SDH_*max*_ per fiber volume and mitochondrial volume density revealed two distinct clusters of DIAm fibers, one comprising type I and IIa fibers and a second comprising type IIx/IIb fibers (Figures 6A,B). Type I and IIa DIAm fibers from both 6- and 24-month rats had higher SDH_*max*_ per fiber volume and higher mitochondrial volume densities as compared with type IIx/IIb fibers of both ages. Type IIx/IIb fibers from 24-month rats had lower SDH_*max*_ per fiber volume and lower mitochondrial volume densities compared with DIAm fibers from 6-month rats. For all DIAm fibers, the SDH_*max*_ per fiber volume was dependent in a positive linear fashion on mitochondrial volume density in both 6-month (slope = 0.21, *P* < 0.0001, *R*^2^ = 0.82) and 24-month rats (slope = 0.17, *P* < 0.0001, *R*^2^ = 0.87), with, the slope being greater in 6-month rat DIAm fibers [*F*_(1,716)_ = 42.9, *P* < 0.0001; Figures 6A,B]. Note that the clustering showed overlap between 6- and 24-month type I and IIa DIAm fibers, but clear separation between type IIx/IIb fibers with age (Figures 6A,B).

### Relation Between Maximum Velocity of the Succinate Dehydrogenase Reaction per Mitochondrial Volume and Mitochondrial Fragmentation

Within an individual DIAm fiber, the relation between SDH_*max*_ per mitochondrial volume and mitochondrial fragmentation (MCI) also revealed two distinct clusters of DIAm fibers, one comprising type I and IIa fibers, and a second comprising type IIx/IIb fibers (Figures 6C,D). Type I and IIa DIAm fibers of both 6- and 24-month rats had higher SDH_*max*_ per mitochondrial volume and higher MCI as compared with type IIx/IIb fibers. In 24-month rats, SDH_*max*_ per mitochondrial volume of type IIx/IIb fibers was similar to 6-month rats, but MCI was lower as compared with DIAm fibers from 6-month rats. Note that the clustering shows overlap between 6- and 24-month type I and IIa DIAm fibers, but clear separation between type IIx/IIb fibers with age (Figures 6C,D).

## Discussion

The novel findings of the present study are that DIAm sarcopenia in 24-month Fischer 344 rats is associated with changes in mitochondrial structure and function in type IIx/IIb fibers. These mitochondrial changes include: (1) reduced mitochondrial volume and volume density; (2) mitochondrial fragmentation; and (3) reduced SDH_*max*_ normalized to fiber volume. In both 6- and 24-month rats, there was a clear relation between SDH_*max*_ per fiber volume and mitochondrial volume density in DIAm fibers, with two distinct clusters, one comprising type I and IIa fibers and the second comprising type IIx/IIb fibers. This distinction was further exaggerated in 24-month rats with a reduction in both SDH_*max*_ per fiber volume and mitochondrial volume density. There was also a clear relation between SDH_*max*_ per mitochondrial volume and mitochondrial fragmentation (MCI), with two distinct clusters of DIAm fibers, one comprising type I and IIa fibers, which were more filamentous, and the second comprising type IIx/IIb fibers, which were more fragmented. This relation also persisted and was exaggerated in 24-month rats with increased mitochondrial fragmentation in type IIx/IIb fibers.

In accordance with past reports in Fischer 344 rats, where colony survival at 24 months old is ∼50% (Cameron et al., 1985; Turturro et al., 1999), the cohort assessed here exhibited marked sarcopenia. The results of the present study showed an ∼29% reduction in DIAm specific force and an ∼35% reduction in the contribution of type IIx/IIb fibers to total DIAm volume. These results are commensurate with previous reports (Gosselin et al., 1994; Elliott et al., 2016; Khurram et al., 2018b; Fogarty et al., 2019b, 2020). In Fischer 344 rats, DIAm sarcopenia is associated with a disproportionate loss of larger phrenic motor neurons (Fogarty et al., 2018b), resulting in denervation, contributing to increased vulnerability to neuromuscular transmission failure (Fogarty et al., 2019a) and impaired maximum transdiaphragmatic pressures (Khurram et al., 2018b). These deficits reflect the reduced ability to perform expulsive and straining maneuvers (Tolep et al., 1995; Polkey et al., 1997; Fogarty and Sieck, 2019a).

### Sarcopenia Is Selective to Type IIx/IIb Diaphragm Muscle Fibers

In the present study, DIAm sarcopenia in Fischer 344 rats was confirmed by the selective atrophy of type IIx/IIb fibers and a reduction in maximum specific force. These results are in agreement with our previous results in male and female Fischer 344 rats (Gosselin et al., 1994; Elliott et al., 2016; Khurram et al., 2018b; Fogarty et al., 2019a,b, 2020), although the ∼35% reduction in cross-sectional areas of type IIx/IIb DIAm fibers observed in the present study was greater than our previous observations, i.e., ∼20–30% reduction in cross-sectional area (Elliott et al., 2016; Khurram et al., 2018b). As a result of the selective atrophy of type IIx/IIb fibers, the relative contribution of these fibers to total DIAm volume was reduced. Previously in the rat DIAm, we found that the maximum specific force of type IIx/IIb fibers is considerably greater than that of type I and IIa fibers (Geiger et al., 1999, 2000, 2001a,b) and is related to a higher concentration of MyHC. With aging, we also found that the total DIAm MyHC concentration is reduced by ∼15% (Elliott et al., 2016). Together, the age-related changes in the relative contribution of type IIx/IIb fibers to mass and the reduced MyHC concentration in type IIx/IIb fibers would limit the ability of the DIAm to perform higher force, airway clearance and expulsive behaviors (Sieck and Fournier, 1989; Khurram et al., 2018b; Fogarty and Sieck, 2019b). It is also important to note that the cross-sectional areas of type I and IIa DIAm fibers were unaffected by aging. Type I and IIa fibers comprise fatigue resistant motor units that are well suited to perform repeated low-pressure necessitating behaviors such as breathing (Sieck and Fournier, 1989; Sieck, 1991, 1995; Khurram et al., 2018b; Fogarty and Sieck, 2019b). With aging, the ventilatory requirements of DIAm force (pressure) generation persist (Khurram et al., 2018b; Fogarty et al., 2019b). In this regard, it should be noted that the residual force of the DIAm after repeated fatiguing activation was not affected by aging, and sufficient to accomplish ventilatory behaviors (Fogarty et al., 2019b).

### Modeling the Effect of Sarcopenia

In the current study, we modeled the effect of sarcopenia and denervation on the fiber type specific contributions to total DIAm isometric specific force, using data determined from type identified single DIAm fibers (Han et al., 1999; Geiger et al., 2000, 2001a). No differences were observed between males and females rats in isometric force generation of the DIAm in past studies using Fischer 344, so these groups were combined for modeling (Khurram et al., 2018b; Fogarty et al., 2019a,b, 2020). This model accounted for the altered relative contributions to DIAm mass and the previously determined ∼37% reduction in the concentration MyHC_2x_ and MyHC_2B_ isoforms (Elliott et al., 2016) and the associated reduction in specific force of type IIx/IIb fibers. Notably, the decline in DIAm specific force observed experimentally with sarcopenia (Gosselin et al., 1994; Elliott et al., 2016; Khurram et al., 2018b; Fogarty et al., 2019b, 2020) was closely approximated using this modeling approach. Thus, changes in MyHC concentration per half sarcomere volume, which we assumed to be exclusive to type IIx/IIb fibers and force per cross-bridge that may be apparent in sarcopenic DIAm fibers (Brenner, 1986; Brenner and Eisenberg, 1986; Sieck et al., 1998), a phenomenon consistent with a selective effect on type IIx/IIb DIAm fibers in our past experiments in denervation (Sieck and Zhan, 2000; Geiger et al., 2001a, 2003; Fogarty and Sieck, 2020).

The ability of the DIAm to sustain ventilation without fatigue is essential to ensure uninterrupted gaseous exchange within the lungs (Belman and Sieck, 1982; Sieck and Fournier, 1989; Sieck, 1991; Fogarty and Sieck, 2019b). The preservation of type I and type IIa fibers in aging is supported by data from the current study, which shows that following 2 min of repetitive activation, the residual specific force generated by the DIAm is unchanged with age, even though the extent of DIAm force fatigue is greater in 6- compared with 24-month rats. Importantly, the improved fatigue index of the DIAm from 24-month rats was entirely due to the lower initial DIAm force, consistent with previous reports in aged Fischer 344 rats (Fogarty et al., 2019b, 2020).

In the current study, we modeled the effect of fatigue on DIAm specific force with aging, using the same determinations of reduced contributions to total mass and reduced contributions of type IIx/IIB fiber specific force as outlined above (Han et al., 1999; Geiger et al., 2000, 2001a; Elliott et al., 2016). In addition, differential fatigability was factored into our assessment, with type I and IIa fibers assumed to have a fatigue index of 0.8, while type IIx/IIb fibers were assumed to have a fatigue index of 0.1, based on previous studies in the cat (Sieck and Fournier, 1989). The modeling of specific forces predicted a residual force following repetitive activation that was greater than that experimentally observed in both the present study and in previous studies (Fogarty et al., 2019b, 2020). Notably, in cases where DIAm fibers were stimulated at lower frequencies (i.e., at 10 Hz) during the fatigue test, the model better predicted the experimentally observed residual forces (Fogarty et al., 2019b). Regardless of the utility of the model, the experimental residual forces generated by DIAm following fatigue are sufficient to provide for the generation of eupneic pressures necessary for ventilation (Fogarty et al., 2019b). The maintenance of function and morphology of type I and IIa DIAm fibers with age is also evident in their maximum respiratory capacities (i.e., SDH_*max*_), which were preserved with aging (Fogarty et al., 2020). We speculate that the difference between modeled and experimentally observed results relates to mitochondrial function in the *ex vivo* environment, which may be impaired at room temperature, as evidenced by the lack of mitochondrial movement at reduced temperatures (Delmotte et al., 2017; Delmotte and Sieck, 2019).

### Mitochondrial Fragmentation in Type IIx/IIb Diaphragm Muscle Fibers Is Exacerbated With Age

Previous observations have shown that mitochondrial morphology may affect their respiratory capacity in a variety of cell types (Vincent et al., 2019; Delmotte et al., 2021). For example, in human airway smooth muscle (hASM), we found that maximum cellular O_2_ consumption rate of mitochondria was higher when mitochondrial morphometry was more filamentous (Delmotte et al., 2021). In another study, mitochondrial morphology was a distinguishing characteristic across muscle fiber types: type I and IIa being more filamentous and type IIx/IIb being more fragmented (Mishra et al., 2015), results similar to those of the present study in the DIAm. In highly active tissues, like the DIAm, mitochondria exhibiting a more filamentous morphology have been suggested to represent the standard state (Trewin et al., 2018; Chaudhry et al., 2020). Filamentous mitochondria have a higher surface area compared with volume ratio, which suggests a greater inner mitochondrial membrane surface area, which would allow for increased O_2_ consumption and oxidative phosphorylation.

In the present study, mitochondrial morphology was assessed in a fiber type specific manner, and the morphology of these individual mitochondria within the DIAm fibers differed across fiber types. Both measurements of mitochondrial morphology (mean branch length and MCI) varied across fiber types. In all type I and IIa DIAm fibers, regardless of age, mitochondrial morphology was more filamentous, with increased mean branch lengths and MCIs. In type IIx/IIb DIAm fibers, regardless of age, mitochondrial morphology was more fragmented, with shorter mean branch lengths and lower MCIs. With age, the fragmentation of mitochondria within type IIx/IIb fibers was exacerbated, with lower branch lengths and lower MCIs compared with type IIx/IIb DIAm fibers from 6-month rats. We propose that these morphological changes in type IIx/IIb DIAm fibers in 24-month rats reflect impaired mitochondrial function, with reduced maximum respiratory capacity (SDH_*max*_ or O_2_ consumption rate) and oxidative phosphorylation due to the reduced surface area of the inner mitochondrial membrane. The mitochondrial morphology of the type I and IIa fibers remains unchanged with age, indicating that maximum mitochondrial respiratory capacity does not change due to their continued activity as required to sustain ventilation. Notably, the maintenance of mitochondrial morphology in aged type I and type IIa fibers is entirely consistent with the resilience of smaller phrenic motor neurons, the preservation of type I and type IIa DIAm fiber cross-sectional areas, unchanged residual forces following fatigue and functional maintenance of eupnea (Elliott et al., 2016; Fogarty et al., 2018b,2019b, 2020; Khurram et al., 2018b).

### Decreased Mitochondrial Volume Density in Type IIx/IIb Diaphragm Muscle Fibers of 24-Month Old Rats

The mitochondrial volume density results of the present study are in general agreement with results in rat DIAm from a previous study from our lab using EM, with type I and IIa fibers having greater mitochondrial volume density than type IIx/IIb fibers (Sieck et al., 1998). In the present study, we provide novel results showing that the absolute volume of mitochondria and mitochondria volume density within type I and IIa DIAm fibers are not affected by age. However, there was a marked decrease in absolute volume of mitochondria and mitochondrial volume density in type IIx/IIb DIAm fibers from 24-month rats when compared with 6-month type IIx/IIb DIAm fibers. Regardless of age, type I and IIa fibers had a greater mitochondrial volume density compared with type IIx/IIb fibers, consistent with their incessant requirements for activation during breathing. Similar to the conservation of mitochondrial structure, the maintenance of mitochondrial volume density in type I and IIa fibers is consistent with their selective resilience to sarcopenia.

### Reduced Maximum Velocity of the Succinate Dehydrogenase Reaction in Type IIx/IIb Diaphragm Muscle Fibers in 24-Month Old Rats

In the present study, both 6- and 24-month type I and IIa fibers in the rat DIAm had higher SDH_*max*_ than type IIx/IIb fibers. This is consistent with past reports in the rat DIAm (Sieck et al., 1989b, 1995; Johnson and Sieck, 1993; Lattari et al., 1997; Zhan et al., 1997; Fogarty et al., 2020). There SDH_*max*_ in type IIx/IIb fibers was markedly lower in DIAm from 24-month compared with 6-month rats, consistent with a past report in Fischer 344 rats (Fogarty et al., 2020). The higher SDH_*max*_ of the type I and IIa fibers is consistent with their frequent activation during ventilation (Sieck and Fournier, 1989; Sieck, 1991; Fogarty and Sieck, 2019b) and their higher mitochondrial volume densities. The substantially lower SDH_*max*_ of type IIx/IIb fibers is consistent with their less frequent activity associated only with expulsive behaviors (Sieck and Fournier, 1989; Sieck, 1991; Fogarty and Sieck, 2019b) and their lower mitochondrial volume densities. Consistent with our previously reported denervation of Fisher 344 DIAm at 24-months (Fogarty et al., 2018b,2019a), our current observation of lower SDH_*max*_ in type IIx/IIb DIAm fibers from 24-month rats is strikingly similar to SDH_*max*_ results from surgically denervated rats (Miyata et al., 1995; Zhan et al., 1997).

### Maximum Velocity of the Succinate Dehydrogenase Reaction per Fiber Volume Is Dependent on Mitochondrial Volume Density

A major innovation of the current study is our novel observations quantifying the relation between SDH_*max*_ per fiber volume and mitochondrial volume density identified two distinct populations of DIAm fibers. Type I and IIa DIAm fibers displayed higher SDH_*max*_ per fiber volume and mitochondrial volume densities compared with type IIx/IIb fibers. This relation between SDH_*max*_ per fiber volume and mitochondrial volume density was unaffected by aging in type I and II DIAm fibers but became more exaggerated in type IIx/IIb fibers with a reduction in both SDH_*max*_ per fiber volume and mitochondrial volume density. We are unaware of any previous study that has explored this basic structure function relation of mitochondria at the single fiber level.

### Maximum Velocity of the Succinate Dehydrogenase Reaction per Mitochondrial Volume Is Dependent on Mitochondrial Fragmentation

In a significant advance from previous approaches, we normalized SDH_*max*_ to mitochondrial volume in individual type identified DIAm fibers and assessed the relation to mitochondrial morphology. Importantly, SDH_*max*_ per mitochondrial volume was significantly higher in type I and IIa DIAm fibers compared with type IIx/IIb fibers, indicating intrinsic differences in the maximum respiratory capacity of mitochondria across fiber types. Strikingly, a unitary mitochondrion in a type I or IIa fiber is capable of ∼4 times the oxidative capacity of a type IIx/IIb fiber. Thus, the higher SDH_*max*_ per fiber volume in type I and IIa DIAm fibers is not due entirely to higher mitochondrial volume density but reflects a higher intrinsic respiratory capacity of mitochondria as well. Notably, the higher intrinsic respiratory capacity of mitochondria in type I and IIa DIAm fibers was unaffected by aging, again consistent with their increased activation with breathing.

When comparing SDH_*max*_ per mitochondrial volume to MCI, two distinct clusters of DIAm were also apparent, one comprising type I and IIa fibers and a second comprising type IIx/IIb fibers. Type I and IIa DIAm fibers from both 6-month and 24-month rats had higher SDH_*max*_ per mitochondrial volume and higher MCI as compared with type IIx/IIb fibers. In type IIx/IIb DIAm fibers from 24-month rats, the SDH_*max*_ per mitochondrial volume was similar to type IIx/IIb fibers from 6-month animals, but the MCI was lower. The relation between SDH_*max*_ per mitochondrial volume and MCI indicated that more filamentous mitochondria in type I and IIa DIAm fibers have a higher maximum respiratory capacity compared with the more fragmented mitochondria in type IIx/IIb fibers. With aging not only is there a reduction in mitochondrial volume in type IIx/IIb fibers, but mitochondria also become more fragmented. These combined factors appear to contribute to the age-associated reduction in SDH_*max*_ per fiber volume in type IIx/IIb DIAm fibers.

In a plethora of conditions, including those involving selective motor neuron and degeneration of fatigable motor units lead to denervated type IIx/IIb skeletal muscle fibers, such as amyotrophic lateral sclerosis (ALS) (Hegedus et al., 2007, 2008; Dukkipati et al., 2018; Fogarty, 2018; Fogarty et al., 2019c,2021a), altered mitochondrial structures and vacuolations accounts for some of the earliest pathology (Wong et al., 1995; Bendotti et al., 2001; Sasaki et al., 2004; Martin et al., 2007; Sasaki and Iwata, 2007; Fogarty et al., 2017; Gautam et al., 2019). Indeed, mutations of mitochondria (i.e., the SOD1 mutant) underpin one of the most widely used (and extensively validated) ALS rodent models (Gurney et al., 1994; Jara et al., 2012; Fogarty, 2018), with mitochondria are a proposed target of recently approved clinical therapies (Takayasu et al., 2007; Writing and Edaravone (MCI-186) ALS 19 Study Group, 2017; Ohta et al., 2020). Consistent with these phenomena, albeit over a more protracted temporal scale, is our observation of increased mitochondrial fragmentation in the type IIx/IIb DIAm fibers from 24-month rats. These type IIx/IIb fibers are likely to be the denervated DIAm fiber population (Fogarty et al., 2018b), and SDH_*max*_ reduction and fragmented mitochondrial morphology data we present, exclusive to type IIx/IIb fibers may be fundamental to their vulnerability to sarcopenia.

## Conclusion

In conclusion, we have assessed and modeled the DIAm in a systematic manner to assess muscle quality, as defined by the capacity for force generation and energy production to support motor unit/muscle fiber type recruitment. These assessments and models show that muscle quality, namely specific force generation, oxidative capacity, mitochondrial abundance, and mitochondrial morphology is perturbed with aging, exclusively in type IIx/IIb fibers. Type I and IIa DIAm fibers remain highly active in sustaining breathing throughout life. Accordingly, aging does not appear to affect mitochondrial structure and function in these fibers. Mitochondria in type I and IIa DIAm fibers are abundant and more filamentous with greater intrinsic respiratory capacity. In contrast, mitochondria in type IIx/IIb DIAm fibers are less abundant and more fragmented with lower intrinsic respiratory capacity. Indeed, mitochondrial fragmentation may be a prime determinant of muscle fiber quality, with aging mitochondria in type IIx/IIb fibers become more fragmented and less functional. Increased fragmentation and dysfunction of mitochondria is a proposed etiology and major pathogenic component of an accelerated condition of age-associated motor neuron loss, ALS. In ALS, efforts to mitigate mitochondrial morphological and functional perturbations have recently met with clinical success. Based on the results of the present study, approaches geared toward ameliorating mitochondrial deficits within these vulnerable fibers may be successful in combating the age-associated decline in skeletal muscle quality.

## Data Availability Statement

The raw data supporting the conclusions of this article will be made available by the authors, without undue reservation.

## Ethics Statement

All protocols were approved by the Mayo Clinic Institute Animal Care and Use Committee (IACUC #A57714) and complied with National Institutes of Health (NIH) and American Physiological Society guidelines.

## Author Contributions

AB, LD, MF, and GS: conceptualization, methodology, formal analysis, investigation, and writing. All authors contributed to the article and approved the submitted version.

## Conflict of Interest

The authors declare that the research was conducted in the absence of any commercial or financial relationships that could be construed as a potential conflict of interest.

## Publisher’s Note

All claims expressed in this article are solely those of the authors and do not necessarily represent those of their affiliated organizations, or those of the publisher, the editors and the reviewers. Any product that may be evaluated in this article, or claim that may be made by its manufacturer, is not guaranteed or endorsed by the publisher.

## Footnotes

1
https://imagej.nih.gov/ij/
